# Regulation of *cry* Gene Expression in *Bacillus thuringiensis*

**DOI:** 10.3390/toxins6072194

**Published:** 2014-07-23

**Authors:** Chao Deng, Qi Peng, Fuping Song, Didier Lereclus

**Affiliations:** 1 State Key Laboratory for Biology of Plant Diseases and Insect Pests, Institute of Plant Protection, Chinese Academy of Agricultural Sciences, Beijing 100193, China; E-Mails: dengchaowin@sina.com (C.D.); qpeng@ippcaas.cn (Q.P.); 2 INRA, UMR1319 Micalis, La Minière, Guyancourt 78280, France; 3 AgroParisTech, UMR Micalis, Jouy-en-Josas 78352 cedex, France

**Keywords:** *cry* gene, transcription, metabolism, crystallization, protein expression, regulation, RNA stability

## Abstract

*Bacillus thuringiensis* differs from the closely related *Bacillus cereus* group species by its ability to produce crystalline inclusions. The production of these crystals mainly results from the expression of the *cry* genes, from the stability of their transcripts and from the synthesis, accumulation and crystallization of large amounts of insecticidal Cry proteins. This process normally coincides with sporulation and is regulated by various factors operating at the transcriptional, post-transcriptional, metabolic and post-translational levels.

## 1. Introduction

**Figure 1:**
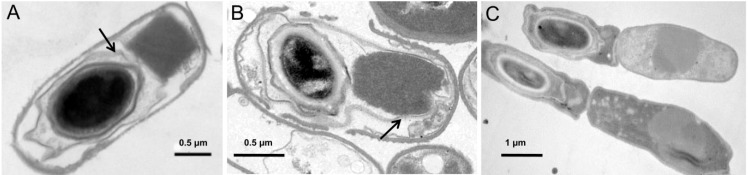
Different patterns of crystal production in Bt. (**A**) Strain HD73 with a typical parasporal crystal phenotype: the crystal is produced beside the spore, in the mother cell compartment; (**B**) Strain YBT-020 with the spore-crystal association phenotype: the crystal is produced between the exosporium and the spore coat; (**C**) Strain LM1212 with the crystal-producer and spore-former differentiation phenotype: crystal and spore are produced in different cell subpopulations. The exosporuium is indicated by arrows.

*Bacillus thuringiensis* (Bt) is a spore-forming bacterium, which is genetically very closely related to *B. anthracis*, the agent of anthrax, and to *B. cereus*, an opportunistic human pathogen that causes food-borne gastroenteritis [1]. One of the most evident differences between Bt and its relatives is its ability to produce crystal inclusions during the stationary phase of growth (Figure 1) [1]. By contrast, the plasmids carrying the toxin genes of *B. anthracis* and *B. cereus* are absent from Bt [2]. The crystals produced by Bt mainly consist of Cry proteins, most of which are toxic for specific insects [3], and consequently Bt has been widely and successfully used as a biopesticide for more than 50 years [3,4]. The crystal inclusion can account for up to 25% of the dry weight of Bt cells, which means that a massive production of proteins should occur during stationary phase (each cell has to synthesize 10^6^ to 2 × 10^6^ δ-endotoxin molecules to form a crystal) [5]. The mechanism for the massive expression of Cry proteins in Bt has been investigated and involves numerous factors: transcriptional regulation, *cry* gene copy number, the stability of *cry* gene mRNA, and the accumulation and crystallization of Cry proteins [5,6]. Many findings relevant to the regulation of *cry* gene expression have been reported over the last 20 years. They indicate that the regulation of Bt *cry* gene expression is more complex than formerly thought: some sporulation-dependent *cry* genes are under the control of the transition-phase sigma factor SigH during the transition phase; some non-sigma factors contribute to the regulation of *cry* gene expression; metabolic pathways may influence Cry protein production; and the description of a unique Bt strain, LM1212, has changed our view of Bt *cry* gene transcriptional regulation and expression patterns. The aim of this review is to consider and collate current knowledge about the regulation of *cry* gene expression in Bt.

## 2. Transcriptional Regulation

The primary regulation of gene expression is at the transcriptional level. The *cry* genes have been classified into two types according to their transcriptional regulation mechanisms: sporulation-dependent *cry* genes are controlled by sporulation-specific sigma factors SigK and/or SigE; and sporulation-independent *cry* genes are under the control of the vegetative SigA factor [5]. Some accessory factors also contribute to the transcriptional regulation of *cry* gene expression (Table 1).

**Table 1:** Transcription factors involved in *cry* gene expression.

Gene	Sigma factors	Other TFs	References
*cry1A*	σ^E^, σ^K^	Spo0A (+); PDH E2 (+)	[7,8,9]
*cry2A*	σ^E^	NA	[10]
*cry2B*	σ^E^	NA	[10]
*cry3A*	σ^A^	NA	[5]
*cry4A*	σ^E^, σ^K^, σ^H^	PPK(+); Hpr/CcpA(−)	[11,12,13,14]
*cry4B*	σ^E^, σ^K^	NA	[11]
*cry6Aa2*	NA	ORF2 (−)	[15]
*cry8Ea1*	σ^E^, σ^K^, σ^H^	NA	[16]
*cry11A*	σ^E^, σ^K^, σ^H^	Spo0A (−)	[11]
*cry15A*	σ^E^	NA	[17]
*cry18A*	σ^E^, σ^K^	NA	[18]

Notes: TFs: Transcription factors; (−): negative regulation; (+): positive regulation. NA: undetermined.

### 2.1. Sporulation-Dependent cry Genes

The sporulation of *Bacillus* species initiates with the asymmetric division of cellular compartment into two parts: the mother cell and the forespore. In the model organism *B. subtilis* (Bs), this process is temporally and spatially regulated by a set of sigma factors of RNA polymerase: the main vegetative sigma factor SigA and SigH in the pre-asymmetric division cell; SigE and SigK in the mother cell; and SigF and SigG in the forespore [19]. Homologous sigma factors (SigA, SigH, SigE, SigK, SigF and SigG) have been found in Bt and play similar roles as those in Bs. Thus, it is generally assumed that the sporulation process in Bt is roughly the same as that in Bs [20,21,22]. Many *cry* genes have been defined as sporulation-dependent because their transcription is mainly controlled by the mother cell-specific sigma factors SigE and SigK.

The transcription of many *cry* genes, including *cry1* [7,23], *cry4* [11,24,25], *cry8* [16], *cry11* [11] and *cry18* [18], is controlled by both SigE and SigK. The transcription is initiated by SigE at the early stage of sporulation and continued by SigK at the late stage of sporulation [20]. The successive activation of these two mother cell-specific sigma factors ensures the continuous and strong transcription of *cry* genes in the mother cells. This allows the production of massive amounts of Cry proteins during sporulation. The transcription of a minority of the sporulation-dependent *cry* genes, notably *cry15A* (formerly known as *cry34*) [17] and *cry2* [10], is controlled by SigE alone. These genes are expressed for a relatively shorter period than *cry* genes directed by both SigE and SigK [21].

Some of the sporulation-dependent *cry* genes, for example, the *cry1* [8], *cry4* [11], *cry8* [16], and *cry11* [11], are weakly expressed at the end of the vegetative growth phase. This expression is initiated by the vegetative sigma factor SigH. The SigH-dependent promoter of *cry1Ac* maps upstream from the SigE- and SigK-dependent promoters [8]; the *cry4* and *cry11* SigH-dependent promoters overlap with the SigE-dependent promoters [11,24]; and the SigH-dependent promoter of *cry8E* maps in the intergenic region between the *cry8E* coding sequence and an upstream gene called *orf1* [16]. Thus, there is not a single model that applies to the transcriptional regulation of all *cry* genes, and their regulation depends on a combination of promoters allowing various expression patterns during sporulation.

### 2.2. Sporulation-Independent cry Genes

The transcription of *cry3* genes is initiated during the vegetative growth, is activated at the end of exponential phase and continues for several hours during the stationary phase [5]. Unlike the sporulation-dependent *cry* genes, which are controlled by sporulation-specific sigma factors, transcription of *cry3* genes is directed by the vegetative SigA promoter and the expression of *cry3* genes is stronger in sporulation-defective *spo0A* and *spo0F* mutants than the wild-type [5,26,27,28]. The *cry3* genes were the only examples of sporulation-independent *cry* genes until the discovery of an unusual Bt strain, LM1212 [29].

**Figure 2:**
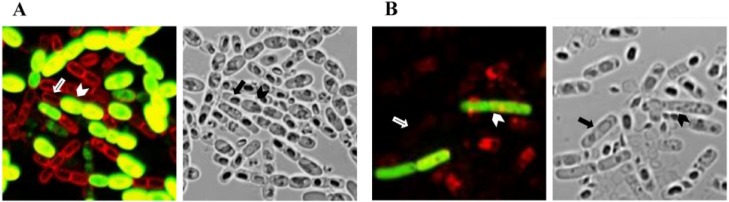
Specific transcription of LM1212 *cry* gene promoter in non-sporulating cells of Bt strains. (**A**) The LM1212 *cry* gene promoter drives transcription specifically and strongly in strain LM1212 in a subpopulation of crystal-producers; (**B**) The LM1212 *cry* gene promoter in strain HD73 drives transcription specifically and weakly in a small non-sporulating subpopulation of cells. Left: GFP/FM^®^-4-64 overlay. Right: bright field image. Sporulating cells are indicated by arrows and GFP-expressing cells are indicated by arrow heads. Cells were grown in SSM medium until T20 with erythromycin. The cell walls were stained with FM^®^-4-64.

Strain LM1212 displays a unique and intriguing phenotype: there is differentiation of crystal-producing cells and spore-forming cells in the population. Thus, crystals are produced in a subpopulation of cells that do not sporulate, and not in the mother cell compartment of sporulating cells (Figure 1). Transcriptional analysis of LM1212 *cry* genes revealed that the promoters of these genes are activated at the end of exponential growth and, in LM1212, are continually expressed for several hours. In sharp contrast, the activity of LM1212 *cry* gene promoters is very low in the wild-type Bt strain *kurstaki* HD73 throughout growth. Work with promoter-*gfp* fusions revealed that the LM1212 *cry* genes are specifically transcribed in a subpopulation of non-sporulating cells in *Bacillus* species, and this subpopulation is much smaller in strains other than LM1212 (Figure 2). The analysis of the expression of the promoter-*gfp* fusion also indicated that the LM1212 *cry* genes are not controlled by the sporulation-specific sigma factors SigE or SigK. Thus the LM1212 *cry* genes are sporulation-independent and are controlled by a novel mechanism of transcriptional regulation.

### 2.3. Regulation of cry Gene Expression by Other Factors

#### 2.3.1. The Transcriptional Regulator Spo0A

The DNA-binding protein Spo0A is the master regulator for entry into sporulation in *B. subtilis* [30]. The phosphorylated form of Spo0A (Spo0A~P) binds to a DNA sequence known as the “0A-box” and acts as both a repressor of certain vegetatively expressed genes and an activator of sporulation-specific genes [30]. The temporal and spatial gene regulation during the sporulation process in Bt is very similar to that in Bs, and the Spo0A proteins of Bt and Bs are homologous [28]. An *in silico* analysis found DNA sequences similar to the “0A-box” upstream from the *cry* genes (*cry4A*, *cry4B* and *cry11A*) in *B. thuringiensis* subsp. *israelensis* (Bti) [11]. The transcription level of the *cry11A* promoter is higher in the *spo0A* mutant than that in the *sigE* mutant [11]. However, deletion of the putative “0A-box” increases expression of the *cry11A* operon, and resulted in its activity being higher in the *sigE* mutant than that in the *spo0A* mutant [11]. These results suggest that the expression of *cry11A* is repressed by Spo0A via its binding to the putative “0A-box” [11]. By contrast, the *cry1Ac* promoter has a higher activity in the *sigE* mutant than in the *spo0A* mutant, suggesting that this *cry* gene is positively regulated by Spo0A during exponential and transition phases [7]. However, expression of the sporulation-specific *cry* genes is very much lower in a *spo0A* mutant than in a wild-type strain. Altogether, these results indicate that Spo0A has different regulatory roles for different *cry* genes, including: (i) a moderate role during the transition phase to modulate *cry* gene expression before the onset of sporulation; and (ii) a major role involving triggering the activity of the sporulation-specific sigma factors.

#### 2.3.2. The E2 Subunit of the Pyruvate Dehygrogenase (PDH) Complex

The PDH complex is widely distributed in bacteria and catalyzes the oxidative decarboxylation of pyruvate to acetyl-coenzyme A, linking the glycolysis pathway with the tricarboxylic acid (TCA) cycle [31]. The PDH E2 subunit in Bs is involved in regulating gene expression during sporulation [32]. In Bt, the PDH E2 subunit specifically binds to sites 200–300 bp upstream from the *cry1A* gene promoter [9]. Mutations of these sites decrease both the binding of the E2 subunit and the transcription of *cry1A* promoter [9]. Thus, the PDH E2 subunit can positively regulate *cry1A* transcription by specifically binding to the 5’ non-coding region of the *cry1A* gene. These observations imply that the PDH E2 subunit may function as a transcription factor, independent of its enzymatic activity. They also imply a link between catabolism and Cry protein synthesis.

#### 2.3.3. Regulation by Two Negative Factors

Yu *et al.* [15] studied a fragment from Bt strain YBT-1518 carrying *cry6Aa2* and its downstream *orf2*, which are co-transcribed, and the inverted repeat sequence found between the two ORFs. The inverted repeat sequence disturbs *orf2* expression [15], probably by down-regulating *orf2* transcription. Over-expression of *orf2* reduced the *cry6Aa2* expression whereas deletion of *orf2* significantly enhanced *cry6Aa2* expression [15]. This work, thus, demonstrates that expression of the *orf2* gene down-regulates the expression of the *cry6Aa2* gene, although the mechanism remains unclear. This is an example of *cry* gene expression being regulated by two negative factors (the inverted repeat sequence and the *orf2* production).

## 3. mRNA Stability

The stability of mRNA is a major determinant of gene expression. The half-life of Bt *cry* genes mRNA is about 10 min, which is significantly longer than the mean half-life of *E. coli* mRNAs (two to three minutes) [33]. The causes of *cry* gene mRNA stability have been discussed elsewhere [5]. However, it is interesting to revisit these mechanisms in the light of recent results.

### 3.1. 3’ Terminal Structure

Inverted repeat sequences are widely found, and are conserved, in the 3’ untranslated regions of many *cry* genes (for example, the *cry1A* genes). They contribute to stabilizing *cry* mRNAs [5,34]. These sequences can form stable stem loop structures which might protect the *cry* mRNA from exoribonucleolytic degradation by 3’-5’ exoribonucleases, which are sensitive to RNA secondary structure [5].

### 3.2. 5’ mRNA Stabilizer

**Figure 3:**
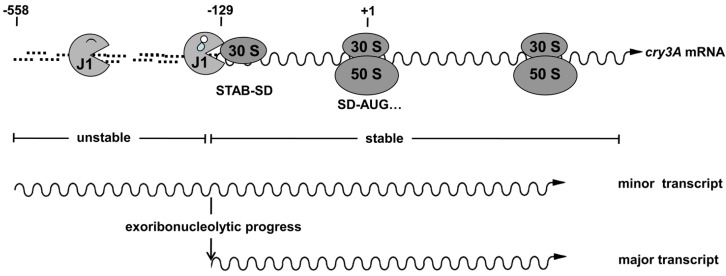
The STAB-SD sequence in cry3A mRNA acts as a 5’ mRNA stabilizer. RNase J1 degrades the −558 transcript of *cry3A* mRNA from 5’ to 3’; the 30S ribosomal subunit can bind to the STAB-SD sequence of *cry3A* mRNA and block the further 5’-3’ exoribonucleolytic progress of RNase J1.

Two transcripts are generated from the cry3A gene: (i) a minor transcript starting 558 bp upstream from the start codon; and (ii) a major transcript starting 129 bp upstream from the start codon [5,35] (Figure 3). A Shine-Dalgarno (SD)-like sequence close to the 5’ of the −129 position is responsible for stabilizing the cry3A mRNA [36]: this sequence (designated as STAB-SD) does not direct translation initiation but can interact with the 3’ end of the 16S rRNA [36]. Therefore, it was suggested that the binding of a 30S ribosomal subunit to the STAB-SD sequence stabilizes the mRNA by preventing degradation by 5’-3’ exoribonuclease [36]. However, it was previously believed that bacteria only possessed mRNA degradation activities that progressed in the 3’ to 5’ orientation. The discovery of the 5’-3’ exoribonuclease activity of the RNase J1 confirmed the original hypothesis: RNase J1 degrades the −558 transcript of cry3A mRNA from 5’ to 3’; the 30S ribosomal subunit binds to the STAB-SD sequence of cry3A mRNA and blocks 5’-3’ exoribonucleolytic progress of RNase J1 (Figure 3) [37]. Similar STAB-SD sequences are also present in the 5’ untranslated regions of other genes (other cry3 genes and genes in Gram-positive bacteria other than Bt) and may therefore be a widespread determinant of mRNA stability.

## 4. Metabolic Regulation of Cry Protein Production

The metabolic pathways that provide carbon components, amino acids, and energy for sporulation and massive synthesis of Cry proteins in Bt are expected to be subject to substantial regulation. Indeed, a recent report reveals metabolic regulation mechanisms involved at both the transcriptional and translational levels [38]: they include controls over the metabolism of proteases and amino acids, the supply of carbon and energy sources, the regulation and modification of metabolic pathways, and the regulation of oxidative phosphorylation and energy generation. The regulation of Cry protein expression via the metabolism-associated sigma 54 factor, and according to polyphosphate metabolism and glucose catabolite repression has been described (see below).

### 4.1. Regulation by the Sigma 54 Factor

The sigma factor sigma 54 (also called SigL) regulates various nitrogen and carbon sources, and energy metabolism pathways in bacteria. We recently found that mutation of sigma 54 in Bt strain HD73 decreased Cry1Ac protein production in LB medium (Peng and coll., unpublished data). The positive effects of sigma 54 on crystal production are at the transcriptional level, and may be indirect involving sigma 54-dependent metabolic pathways. For example, the γ-aminobutyric acid (GABA) shunt, which is controlled by sigma 54 and regulated by GabR [39], is an additional supplement to the TCA cycle and was shown to be correlated with spore and parasporal crystal formation in *B. thuringiensis* [40]. Accordingly, the metabolism of GABA becomes more active during sporulation [38]. Another example is the *acoABCL* operon encoding the acetoin dehydrogenase complex [41]: *acoABCL* is strongly up-regulated at both the transcriptional and translational levels during sporulation [38]. We confirmed that this operon is controlled by sigma 54 and is activated by AcoR (Peng and coll., unpublished data). However, sigma 54 does not affect Cry1Ac transcription and production in sporulation medium (SSM), indicating that the effects of metabolic pathways on Cry protein production depend on growth conditions.

### 4.2. Polyphosphate Kinase (PPK) and Polyphosphate Metabolism

Some mineral nutrients, especially inorganic phosphate, are important for Cry protein production in Bt [42,43,44]. Polyphosphate is synthesized by PPK from the terminal phosphate of ATP [45] and can be degraded to inorganic phosphate by endopolyphosphatase and exopolyphosphatase enzymes [46]. Doruk *et al.* (2013) reported that the overexpression of PPK in Bti increased both the intracellular concentration of polyphosphate and Cry protein production. They also found that the transcription of *sigE* was stimulated in the strain overexpressing PPK [12]. Thus, changes to polyphosphate metabolism may influence Cry protein production in Bt, probably via SigE.

### 4.3. Catabolic Repression by Glucose

In Gram-positive bacteria, catabolite repression of many catabolic operons involves the phosphocarrier protein HPr, the catabolite control protein CcpA, and a *cis*-acting catabolite responsive element (*cre*) [13,14]. The phosphorylated HPr binds to the CcpA to form a complex with strong DNA binding affinity [47,48]. The phosphorylated HPr-CcpA complex modulates the transcription of target genes by binding to the *cre* sequence [49]. Glucose represses *cry4A* gene expression at the mRNA level in Bti [50], and the phosphorylated HPr-CcpA complex of Bti represses *cry4A* transcription by specifically binding to a 15 bp *cre* sequence overlapping the -35 element of the *cry4A* promoter [14]. Glucose catabolite repression of *cry4A* is abolished both by site-specific mutation of the *cre* sequence [14] and by the HPr-S45A mutant, which produces phosphorylation-disabled HPr [13]: both increase the activity of the *cry4A* promoter. Thus, in Bti, the synthesis of Cry protein is controlled by HPr-CcpA-mediated glucose catabolite repression.

## 5. Crystallization of the Cry Proteins

The accumulation of large amounts of proteins requires production of stable proteins or, at least, a mechanism to prevent degradation of the proteins produced. Bt mainly produces Cry proteins during the stationary phase, when large amounts of various proteolytic enzymes are synthesized. This means that Bt must have mechanisms to prevent proteolysis of the synthesized Cry proteins. One strategy is to form crystals resistant to proteolytic enzymes.

The mass of many Cry proteins (for example, the Cry1 proteins) is between 130 and 140 kDa. Most of these large Cry proteins can spontaneously form crystals independent of the host bacterium; the genes encoding these proteins are generally monocistronic [5,51]. The C-terminal halves of these proteins are called crystallization domains as they are not involved in toxicity but are necessary for the formation of the crystal. Many other Cry proteins of smaller mass, for example, the Cry2, Cry11 and Cry19 proteins, have no C-terminal crystallization domain like that in the 130–140 kDa Cry proteins. The massive accumulation or crystallization of these Cry proteins generally requires the presence of additional proteins encoded by genes in the same operon [5,51]. These additional proteins are in many cases small, have no insect toxicity and are not the main components of the crystals; rather, they enhance the accumulation or crystallization of their accompanying Cry proteins. Consequently, they are described as accessory proteins or helper proteins. These helper proteins include the 19 kDa P19 and 20 kDa P20 proteins encoded by the *p19* and *p20* genes in the *cry11Aa* operon [25]; the 20 kDa Orf1 and 29 kDa Orf2 proteins encoded by the *orf1* and *orf2* genes in the *cry2A* operon [10]; and the 60 kDa ORF2 (60 k) protein encoded by the *orf2* gene of the *cry19A* operon [51].

The function of P19 and Orf1 in Cry protein production and crystallization is not clear. Orf2 is necessary for the crystallization of Cry2A [52,53]. It contains 11 tandem repeats of a 15/16 amino acid motif that is acidic in nature [10,52]. Orf2 and Cry2A can be co-precipitated, evidence of interaction between the two proteins [53]. Indeed, Orf2 serves as a crystallization factor by interacting with the Cry2A protein, possibly acting as a template or scaffold [52,53]. ORF2 (60 k) encoded by the *orf2* of the *cry19A* operon is very similar to the C-terminal domain of the 130 kDa Cry proteins and is essential for the crystallization of Cry19A, which is itself similar to the N-terminal domain of the large Cry proteins [51]. There is various evidence that ORF2 (60 k) functions primarily as the C-terminal crystallization domain of the large Cry proteins: (i) there are approximately equimolar amounts of ORF2 (60 k) and Cry19A in the crystals; (ii) in-frame fusion of Cry19A to ORF2 (60 k) forms stable crystals; (iii) in-frame fusion of the N-terminal region of Cry1C to ORF2 (60 k) results in the formation of visible inclusions; and (iv) in-frame fusion of Cry19A to the C-terminal crystallization region of Cry1C results in a protein that forms crystals [51]. Similar gene organizations are also found in other *cry* operons such as the *cry10Aa*, *cry39Aa* and *cry40Aa* operons: the upstream reading frame encodes the Cry N-terminal domain, and the frame found about 100 bp downstream encodes a protein similar to the Cry C-terminal crystallization domain [51,54].

The P20 protein can improve the yield and/or crystallization of a variety of insecticidal crystal proteins including Cyt1Aa [55], Cry1Ac [56], Cry2Aa [52], Cry4Aa [57], Cry11Aa [58] and truncated Cry1C [59,60]. It can also enhance the expression of the Bt vegetative insecticidal protein Vip3A [61] and *B. sphaericus* binary toxins BinA-BinB [62]. The effect of P20 on Cyt1A expression was addressed by a series of studies in *E. coli* by Whiteley’s group decades ago: the P20 protein does not affect transcription or mRNA stability [63], nor regulate translational initiation of *cyt1A* [64]; there is a protein-protein interaction between P20 and Cyt1A, and this occurs only with the nascent Cyt1A peptide [65]; P20 is not required for production of large amounts of Cyt1A in *E. coli* strains defective in proteolytic activity [65]. These findings suggest that P20 acts as a molecular chaperone, which helps Cyt1A to form protease-resistant crystals. Another study supports this possibility and provides more details [56]: in Bt, the Cry1Ac protein is substantially degraded throughout the production process, especially during protein synthesis, before crystallization [56]. The introduction of P20 significantly improves Cry1Ac production, and this was the consequence of protection of the nascent peptides [56]. The *p20* gene has been detected in various Bt serovar strains, suggesting that P20 may be a widespread factor influencing the Cry protein crystallization.

## 6. Patterns of Crystal Production

The Cry protein inclusion of Bt is called a parasporal crystal because it is generally produced beside the spore, in the mother cell. This phenotype is mainly determined at the transcriptional level. The sporulation-dependent *cry* genes, mostly controlled by the mother cell-specific SigE and SigK, are transcribed exclusively in the mother cell of sporulating cells, thus restricting the production of crystals to this compartment (Figure 1A). The *cry3* genes, which are under direction of SigA-like promoters, may also be expressed in a subpopulation of non-sporulating cells, although this has never been reported, except for when the *cry3* gene is cloned in a *spo0A* mutant [28].

The discovery and characterization of an unusual strain, LM1212, expanded our understanding of the parasporal crystal phenotype in Bt. In this strain, the crystal is not produced in the mother cell of sporulating cells, but only in a subpopulation of non-sporulating cells (Figure 1C) [29]. The separation of crystal and spore into different LM1212 cell populations is a consequence of cell differentiation associated with a previously undescribed *cry* gene transcription pattern; indeed, the LM1212 *cry* genes are transcribed only in a subpopulation of non-sporulating cells, called crystal-producer cells (Figure 2).

In most Bt strains, crystals separate from spores after lysis of the mother cell. However, a few Bt strains display a spore-crystal association (SCA) phenotype in which the crystals are produced between the exosporium and the spore coat (Figure 1B) [66]. The SCA phenotype is typically found among the Bt subsp. *finitimus* strains. Debro *et al.* [67] reported that genes on a 98 MDa plasmid are responsible for the SCA phenotype in one of these strains. Zhu *et al.* [66] found that the genes necessary and sufficient for the SCA phenotype in another Bt subsp. *finitimus* strainYBT-020 are harbored by two different plasmids: the *cry26Aa* gene on a 188 kb plasmid and a five-gene cluster on a 139 kb plasmid. However, the genome sequence of a Bt strain displaying a SCA phenotype does not contain genes homologous to any of these genes. Therefore, genes determining the SCA phenotype appear to differ among subspecies, or have not so far been correctly identified [66]. Although the biology of the SCA phenotype remains unclear, a tight association between crystal and spore may reduce the risk of the insecticidal toxins being exploited by other microorganisms that do not produce the crystal.

## 7. Concluding Remarks

Bt is the most widely and successfully used bio-insecticide, so agronomic applications are always to the forefront in any considerations of the species. A powerful strategy for improving the use of Bt-based biopesticides is to enhance Cry protein production. This requires a good understanding of the regulation of *cry* gene expression. Indeed, with the characterization of *cry* gene expression mechanisms, a variety of strategies have been used to improve the Cry protein yield from Bt strains: using strong promoters and increasing the *cry* gene copy number [68,69], introducing the STAB-SD sequence [70,71], using the *cry3A* promoter in a sporulating-defective mutant [28], co-expression of a helper protein [56], and using combinations of strategies [72,73]. Another way to extend agronomic applications of Bt is to broaden its insecticidal spectrum. One approach to this is the co-expression of different Cry toxins under the control of different types of *cry* promoters (sporulation-dependent and -independent), an approach that may also increase total Cry protein amounts because competition for regulation factors and nutrition between these promoters and genes is reduced [74,75,76]. We believe that new Bt-based biopesticides could be developed by using novel regulation mechanisms. A better understanding of the mechanism of Cry protein crystallization may facilitate the development and synthesis of stable and biologically active crystal inclusions. This has already been demonstrated in the case of the Vip3 toxin: this soluble protein exported in the extra cellular medium during growth can be specifically produced and accumulated in the mother cell of Bt as crystal inclusions by placing an in-frame fusion of the gene with the sequence encoding the C-terminal of *cry1C* under the control of a sporulation-dependent *cry* gene promoter [77].

The expression of *cry* genes by Bt is regulated in a sophisticated way, through mechanisms from the *cry* gene copy number to the involvement of helper proteins and at various levels from transcriptional to metabolic. These strategies, and the variety of *cry* genes, make Bt an efficient biological weapon for killing a wide range of susceptible insects in various ecological niches. This explains why Bt has become the most successful and widely distributed biopesticide in the world. However, it is not clear why during evolution Bt has maintained or developed different *cry* gene expression systems and crystal localization patterns rather than a single, powerful system. The production of Cry toxins in Bt has been used as a model system to study the cooperation and evolutionary ecology of bacterial virulence [78,79]; the division of labor between crystal-producers and spore-formers in LM1212 has been shown to benefit the population when competing with a typical Bt strain [29]. This illustrates how a better knowledge of the mechanisms of *cry* gene expression and crystal localization in Bt can provide important new insights into basic issues and questions of fundamental importance.
